# *Leptospira* spp. and *Rickettsia* spp. as pathogens with zoonotic potential causing acute undifferentiated febrile illness in a central-eastern region of Peru

**DOI:** 10.1186/s13104-024-06837-1

**Published:** 2024-06-20

**Authors:** Wilmer Silva-Caso, Miguel Angel Aguilar-Luis, Walter Espinoza-Espíritu, Mercedes Vilcapoma-Balbin, Luis J. Del Valle, Erika Misaico-Revate, Fernando Soto-Febres, Giancarlo Pérez-Lazo, Johanna Martins-Luna, Francisco Perona-Fajardo, Juana del Valle-Mendoza

**Affiliations:** 1 School of Medicine, Research Centre of the Faculty of Health Sciences, Universidad Peruana de Ciencias Aplicadas, Lima, Peru; 2 Facultad de Medicina Humana, Unidad de Post Grado, Universidad de San Martín de Porres, Lima, Peru; 3 Red de Salud Leoncio Prado. Ministerio de Salud, Huánuco, Peru; 4 Centre d’Enginyeria Biotecnologica i Molecular (CEBIM), Departament d’Enginyeria Quıímica, ETSEIB, Universidad Politécnica de Catalunya (UPC), Barcelona Tech, Spain; 5 Hospital de Tingo María, Ministerio de Salud del Peru, Huánuco, Peru; 6 Division of Infectious Diseases, Guillermo Almenara Irigoyen National Hospital-EsSalud, Lima, 15033 Peru; 7 Instituto de Investigación Nutricional, Lima, Peru

**Keywords:** *Rickettsia*, *Leptospira*, Fever

## Abstract

**Objetive:**

this study was to determine the relationship between acute febrile illness and bacterial pathogens with zoonotic potential that cause emerging and re-emerging diseases in a central-eastern region of Peru.

**Results:**

Out of the 279 samples analyzed, 23 (8.2%) tested positive for infection by *Rickettsia* spp., while a total of 15 (5.4%) tested positive for *Leptospira* spp. Women had a higher frequency of infection by *Rickettsia* spp., with 13 cases (53.3%), while men had a higher frequency of infection by *Leptospira* spp., with 10 cases (66.7%). The most frequently reported general symptom was headache, with 100.0% (*n* = 23) of patients with *Rickettsia* (+) and 86.7% (*n* = 13) of patients with *Leptospira* (+) experiencing it. Arthralgia was the second most frequent symptom, reported by 95.6% (*n* = 22) and 60% (*n* = 9) of patients with *Rickettsia* (+) and *Leptospira* (+), respectively. Myalgia was reported by 91.3% (*n* = 21) and 66.7% (*n* = 10) of patients with *Rickettsia* (+) and *Leptospira* (+), respectively. Retroocular pain, low back pain, and skin rash were also present, but less frequently. Among the positives, no manifestation of bleeding was recorded, although only one positive case for *Leptospira* spp. presented a decrease in the number of platelets.

## Introduction

The acute febrile illness (AFI) usually presents in primary care establishments. In tropical and subtropical regions, it is associated with viral, parasitic, or bacterial causes [1, 2]. Due to its wide spectrum of differential diagnostics and lack of specific tests or multi-pathogen diagnostics platforms at the health care establishment, a proper diagnosis cannot be achieved, which leads to an empirical treatment [3, 4]. Among AFI related agents, bacterial pathogens are included, and more importantly to public health, emerging and re-emerging bacteria involved in new outbreaks of infectious disease which, in the last 20 years, have led to epidemic potential events [2, 5, 6].

In Peru, *Bartonella* spp., *Leptospira* spp., *Rickettsia* spp. has been described as causing AFI in the southern jungle, with a frequency of isolation higher than 30% [7].

Leptospirosis is a zoonotic bacterial disease that presents complex transmission dynamics and epidemiology. In humans it is transmitted through direct contact with the urine of infected domestic or wild animals and exposure to contaminated soil and water [8, 9]. This demonstrates its importance from the point of view of public health, agricultural practices, the need for a vaccine, among other factors of interest focused on One Health for its control and prevention [10].

Infections caused by *R. rickettsii* often lead to severe illness and patients often require hospitalization. Up to 20% of untreated cases and 5% of treated cases can have a fatal outcome [11, 12]. The transmission chain involves the host and the arthropod in an environmental context, which is why it is complex and implies a constant challenge in the prevention and control of the disease [13–16].

*Bartonella* spp. they are common hemopathogens of mammals; two species cause infections of public health importance. Trench fever, caused by *B. quintana* and transmitted by lice, affected hundreds of thousands of soldiers or displaced persons during World War I and affects homeless people to this day. Oroya fever (and its chronic manifestation, Peruvian wart), caused by *B. bacilliformis* and transmitted by sandflies, is a potentially serious febrile illness. Although it is geographically restricted to the altitudes of the Andes and affects a relatively small number of people, it has a high fatality rate without treatment [17].

In the study region Leptospitosis has been reported in the context of agricultural activity [18]. *Bartonella* currently presents epidemiological silence [19]. Rickettsiae are transmitted through a person’s skin. Transmission of spotted fever group species - SFG occurs during feeding on an infected tick. Typhus group organisms are transmitted by inoculation of feces from infected lice or fleas (*R. prowazekii* and *R. typhi*, respectively) into a bite wound or mucous membranes. *Rickettsia akari* is transmitted by the mites *Liponyssoides sanguineus* [20]. Therefore, their identification would allow the development and implementation of specific surveillance and treatment protocols [21–24].

The diagnostic challenge arises in the context of acute febrile illness that represents undifferentiated fevers where it is essential to determine the etiologies in each region. In this context, in previous studies it was determined that dengue viruses caused 14.6% of the cases, the Venezuelan equine encephalitis virus caused 2.5%, the Oropouche virus 1.0%, the Mayaro virus the 0.4% and other arboviruses caused 0.2% of AFI cases. Additionally, 22.9% tested positive for malaria and 9% had evidence of acute leptospirosis [25].

The objective is to understand the role of *Leptospira sp*., *B. bacilliformis* and *Rickettsia sp*. in the etiology of undifferentiated acute febrile illness in a central-eastern region of Peru. This study complements our previous research in other regions of Peru.

## Materials and methods

Based on an observational, descriptive, comparative, cross-sectional study, a secondary database was used, built with the analysis of blood samples from 279 patients with AFI evaluated in the health establishments of the Leoncio Prado Health Network in the year 2016. Samples were obtained in the context of the Supreme Decree N° 014-2016-SA, which declared a health emergency state at the Huánuco department due to an AFI outbreak and in the context of epidemiologic surveillance as a mandatory public health activity. Clinical data were collected by the treating physician of each participating health facility where a standardized format was used for data collection.

### Place of study

Leoncio Prado is a province in the central-eastern jungle of Peru. It has characteristics of a certain tropical ecological homogeneity, with a predominance of humid forest. The minimum annual average temperature is 18.7ºC and the maximum is 30.5ºC. Regarding precipitation, the accumulated annual average is around 3472.8 mm with a relative humidity of 77.5% [26].

### Selection criteria

In the context of epidemiologic surveillance of febrile illness, were included all patients who attended outpatient health facilities with acute febrile illness, defined as an axillary temperature greater than or equal to 38 °C within at least 7 days prior to the consultation with no identifiable source of infection. Conditionally, they may present symptoms associated with fever such as: headache, myalgia, eye pain, joint pain, fatigue, cough, nausea, vomiting, dizziness, throat pain, dyspnea, rhinorrhea, diarrhea, sensory disturbances, jaundice, neck stiffness and bleeding manifestations. Patients with fever and an identifiable focus were excluded.

The importance of other pathogens such as *Coxiella burnettii*, *Borrellia* and other *Bartonella* species, which present a similar clinical condition, is recognized. However, our analysis considers only *Rickettsia*, *Leptospira* and *B. bacilliformis* due to their epidemiological, clinical and public health importance in the region studied based on their potential to represent a serious threat to human and animal health on the fact that that these three bacterial pathogens were subject to the national epidemiological surveillance system for a certain time. The samples were obtained during medical care.

### Laboratory techniques and procedures

#### DNA extraction

Bacterial DNA extraction was performed according to a commercial extraction kit (High Pure, Roche Applied Science, Mannheim, Alemania), with 200 µL of the serum samples. The resulting DNA was eluted in 100 µl of nuclease free water and then it was processed and stored at -20 °C until its usage.

#### PCR amplification

Detection by real-time PCR assay of *Bartonella bacilliformis*, *Leptospira* spp. and *Rickettsia* spp. Amplification of the genetic material was performed using specific primers and a genetic probe specific for Bartonella bacilliformis [27], the PanR8 gene of *Rickettsia* spp [28]. and the LipL32 gene from *Leptospira* spp [29].

The primers and probes used in the identification of pathogens are genus and/or species specific as described below:

For *Bartonella bacilliformis*, Bb gene, with the primers Bb2F (5′-CAATTATCATCATTATTTGC TCCTGG-3′), Bb2R (5′-TACTGCTGAGG TTGGGCGA-3′) and the BbT probe (FAM-AGAAGACG ATCCGTTACAT-MGB) to amplify 117 bp of the fragment of the target gene.

For *Rickettsia* spp., PanR8 gene, with primers PanR8_F (5′-AGC TTG CTT TTG GAT CAT TTG G-3′), PanR8_R (5′-TTC CTT GCC TTT TCA TAC ATC TAG T-3′) and the probe PanR8_P (Fl-CCT GCT TCT ATT TGT CTT GCA GTA ACA CGC CA-BHQ1).

For *Leptospira* spp., LipL32 gene, with the primers LipL32-45 F (5′-AAG CAT TAC CGC TTG TGG TG-3′), LipL32-286R (5′-GAA CTC CCA TTT CAG CGA TT-3′) and the probe LipL32-189P (FAM-5′-AA AGC CAG GAC AAG CGCCG-3′-BHQ1) to amplify 242 bp.

qPCR conditions were 95 °C for 2 min, 55 cycles of 3 s at 95 °C, 30 s at 55 °C, and 10 s at 72 °C. For *B. bacilliformis*, the recollection strain (CIP 57.19, NCTC 12,135) was used as a positive control, while *Leptospira noguchii* y *Rickettsia typhi* strains provided by the Nutritional Research Institute, Lima, Peru, were used as positive controls for *Leptospira* spp. and *Rickettsia* spp. PCR without a DNA template was used as negative control in all the cases. For internal control purposes, a PCR targeting the codifying gen for human beta-globin was included to reject a possible inhibition of PCR caused by inhibitory molecules still present in the sample after undergoing DNA extraction and purification.

## Data processing and analysis

### Statistical analysis

Data analysis was performed with the information obtained and registered in a database. During the initial study, an Excel database was built under the double-entry method to minimize errors. Then, STATA 11.2 version (Data Analysis and Statistical Software) was used. Fisher´s exact test was used to estimate differences statistical (p < = 0.05). The 95% confidence interval was estimated for each frequency or odds and two frequencies or odds were compared with the odds ratio.

### Ethical aspects

Secondary analysis as of a first study previously evaluated and approved by the Ethics Committee of the “Hospital Regional Docente” of Cajamarca due to its multi-centric character, with the file number 1,958,851. Also, it has the execution permit for research studies corresponding to the Health Network and the establishment where the N° 020-2026-GRHCO-HTM-UADI study was performed.

Samples were obtained under a sanitary emergency context due to febrile illness and as a mandatory public health strategy for epidemiologic surveillance, as the guidelines of the World Health Organization (WHO) and the Council for International Organizations of Medical Sciences (CIOMS) determined.

The database is anonymous, no personal identifiers were registered to guarantee the data anonymity and confidentiality.

## Results

Results showed that, of a total 279 analyzed samples of AFI patients, 125 (44.8%) ranged between 18 and 39 years old, followed by 44 (15.8%) patients aged 40 to 59 years old.

In a single case, age was not registered, but it was included for analysis as it resulted positive for *Leptospira* spp. infection.

Of the total samples, 23 (8.2%) were positive for *Rickettsia* spp., of these positive samples, 9 (39%) were between 18 and 39 years old. The samples positive for *Leptospira* spp., in total were 15 (5.4%), of which 6 (40%) were between 18 and 39 years old. Regarding the sex of the patients, 142 (50.9%) were men. For their part, women showed a higher frequency of *Rickettsia* spp. 13 positive (53.3%), while in *Leptospira* spp. 10 (66.7%) were men (Table 1). No sample was positive for *Bartonella bacilliformis.*

**Table 1:** Population characteristics

Characteristics	AFI Total			*Rickettsia* spp +				*Leptospira* spp +			
	**n = 279**	**%**		**n = 23**	**%**	**CI 95%**	**OR**	**n = 15**	**%**	**CI 95%**	**OR**
Age (years)											
<5	16	5.7		0	0	0–14.3	0	0	0	0-20.4	0
5–11	38	13.6		3	13.0	4.5–32.1	0.95	4	26.6	10.8–51.9	2.31
12–17	42	15.1		5	21.8	9.7–41.9	1.57	3	20.0	7.04–45.2	1.41
18–39	125	44.8		9	39.2	22.1–59.2	0.79	6	40.0	19.8–64.3	0.82
40–59	44	15.8		3	13.0	4.5–32.1	0.80	0	0	0–20.4	0
>60	13	4.7		3	13.0	4.5–32.1	3.07	1	6.7	1.1–29.8	1.46
No registered	1	0.3		0	0	-	-	1	6.7	-	-
Sex											
Male	142	50.9		10	43.5	25.6–63.2	0.74	10	66.7	41.7–84.8	1.93
Female	136	48.7		13	56.5	36.8–74.4	1.37	5	33.3	15.2–58.3	53.6
No registered	1	0.4		0	0	-	-	0	0	-	-

Regarding the clinical presentation, among the patients who tested positive for *Rickettsia* spp. or *Leptospira* spp. The most frequent general symptom for *Rickettsia* (+) cases was headache, with a frequency of 100.0% (*n* = 23), followed by arthralgia 95.6% (*n* = 22) and myalgia with 91.3% (*n* = 21). For *Leptospira* (+) cases, headache occurred with a frequency of 86.7% (*n* = 13), followed by arthralgia 60% (*n* = 9) and myalgia with 66.7% (*n* = 10). Retro-ocular pain, skin eruptions, and odynophagia were more frequent in *Rickettsia* (+) patients than in *Leptospira* (+) patients. The hemorrhagic manifestations did not appear in any positive case. Only one patient positive for Leptospira spp. showed decreased platelet count (Table 2).

**Table 2:** Clinical characteristics of patients with positive results for Rickettsia spp. and Leptospira spp

Signs or symptoms		AFI Total		Rickettsia spp. +				Leptospira spp +			
		*n* = 279	%	*n* = 23 (%)	CI 95%		OR	*n* = 15 (%)	CI 95%		OR
General											
	Arthralgia	229	82.1	22 (95.6)	79–99.2		4.80	9 (60.0)	35.7–80.2	0.33	
	Myalgia	231	82.8	21 (91.3)	73.2–97.6		2.18	10 (66.7)	41.7–84.8	0.42	
	Headache	252	90.3	23 (100.0)	85.7–100		-	13 (86.7)	62.1–96.3	0.69	
	Retro-ocular pain	172	61.7	14 (60.9)	40.8–77.8		0.97	5 (33.3)	15.2–58.3	0.31	
	Lumbar pain	127	45.5	9 (39.1)	22.2–59.2		0.77	3 (20.0)	7.0–45.2	0.29	
	Cutaneous eruption	71	25.5	8 (34.8)	18.8–55.1		1.56	2 (13.3)	3.7–37.9	0.45	
	Hyporexia	147	52.7	12 (52.2)	32.9–70.8		0.98	7 (46.7)	24.8–69.8	0.79	
	Odynophagia	80	28.7	4 (17.4)	6.9–37.1		0.52	1 (6.7)	1.2–29.8	0.18	
	Nausea/Vomiting	134	48.0	12 (52.2)	32.9–70.8		1.18	9 (60.0)	35.7–80.2	1.62	
Bleeding manifestations	Hematemesis	6	2.2	0 (0.0)	0–14.3		0	0 (0.0)	0–20.4	0	
	Mane	2	0.7	0 (0.0)	0–14.3		0	0 (0.0)	0–20.4	0	
	Epistaxis	4	1.4	0 (0.0)	0–14.3		0	0 (0.0)	0–20.4	0	
	Gingivorrhagia	3	1.1	0 (0.0)	0–14.3		0	0 (0.0)	0–20.4	0	
	Vaginal bleeding	2	0.7	0 (0.0)	0–14.3		0	0 (0.0)	0–20.4	0	
	Petechiae	4	1.4	0 (0.0)	0–14.3		0	0 (0.0)	0–20.4	0	
	Ecchymosis	1	0.4	0 (0.0)	0–14.3		0	0 (0.0)	0–20.4	0	
	Hemoptoic sputum	3	1.1	0 (0.0)	0–14.3		0	0 (0.0)	0–20.4	0	
Alarm signs	Abdominal pain	12	4.3	0 (0.0)	0–14.3		0	0 (0.0)	0–20.4	0	
	Chest pain	3	1.1	0 (0.0)	0–14.3	0		0 (0.0)	0–20.4	0	
	Persistent vomiting	1	0.4	0 (0.0)	0–14.3	0		0 (0.0)	0–20.4	0	
	Hypothermia	1	0.4	0 (0.0)	0–14.3	0		0 (0.0)	0–20.4	0	
	Decreased diuresis	2	0.7	0 (0.0)	0–14.3	0		0 (0.0)	0–20.4	0	
	Excessive fatigue	1	0.4	0 (0.0)	0–14.3	0		0 (0.0)	0–20.4	0	
	Hepatomegaly	2	0.7	0 (0.0)	0–14.3	0		0 (0.0)	0–20.4	0	
	Decreased platelets	8	2.8	0 (0.0)	0–14.3	0		1 (6.7)	1.2–29.8	2.41	
	Increased hematocrit	2	0.7	0 (0.0)	0–14.3	0		0 (0.0)	0–20.4	0	
	Altered mental status	2	0.7	0 (0.0)	0–14.3	0		0 (0–0)	0–20.4	0	
Shock signs	Hypotension	1	0.4	0 (0.0)	0–14.3	0		0 (0.0)	0–20.4	0	

Statistically significant associations between clinical severity parameters and identification of *Rickettsia* spp. (*p* = 0.146) and *Leptospira* spp. (*p* = 1.001) were not found. (Table 3).

**Table 3:** Correlation between clinical severity and the presence of bacterial pathogens

		*Rickettsia* spp. n (%)		Total *n* = 279 (%)	p*
		Negative 256 (91.8)	Positive 23 (8.2)		
Severity criteria	Present	29 (10,4)	0 (0,0)	29 (10,4)	0.146
	Not present	227 (81.4)	23 (8.2)	250 (89.6)	

		***Leptospira spp***. n (%)		Total *n* = 279 (%)	
		Negative 264 (94.6)	Positive 15 (5.4)		
Severity criteria	Present	28 (10.0)	1 (0.4)	29 (10.4)	1.001
	Not present	236 (84.6)	14 (5.0)	250 (89.6)	

*Fisher´s exact test; p < = 0,05

## Discussion

The results identified two of the three bacterial pathogens tested. Both bacteria have a zoonotic nature and established interaction of pathogen-host-environment [30, 31]. Molecular identification of these pathogens with zoonotic potential requires a well-equipped laboratory, adequate infrastructure, biosafety, and highly trained laboratory personnel, complementing One Health’s integrated approach to the complex relationship between humans, animals, and the environment [32].

In routine clinical practice it is difficult to clearly differentiate the etiology of fever when diagnostic capabilities are limited. This can result in inappropriate and un-timely handling of the patient [33]. In this context, the inappropriate use of antimicrobial drugs can also be highlighted, which implies the development of resistance to antibiotics and a negative impact on the microbiota [34]. On the other hand, it has also been described that it is possible to provide empirical treatment with doxycycline applied to a specific population that consists of travelers who return with undifferentiated fever and negative tests for malaria and dengue, when they present severe illness, factors predictive of rickettsiosis or without characteristics. of dengue. This is in relation to its results similar to those found in our study for *Rickettsia* spp. and *Leptospira* spp [4].

In the case of *Leptospira*, the Ministry of Health conducts epidemiological surveillance in the study region and reports an average of 56.4 (SD = 37.7) confirmed cases per year between 2016 and 2022 [35]. On the other hand, a study carried out in the north coast of the country, identified 36 cases through a microagglutination test, also finding a higher frequency in the young population group [36, 37]. In Latin America, a review by Moreira et al. in 2018 identified *Leptospira* spp. as a common pathogen causing acute febrile illness reported in 5 of 17 studies evaluating 13,539 people [2]. In Brazil, based on a microscopic agglutination test the prevalence of anti-Leptospira antibodies was 1.17% (4/341; CI 0.46-2.98%) [38]. The present work identifies *Leptospira* spp. in 5.4% (15/279; CI 2.21-4.25%) of the individuals evaluated, this percentage is equivalent to that reported in other studies carried out in different regions [2]. However, PCR and MAT results cannot be compared, especially in tropical or endemic areas. In addition, after 7 days post-infection, analysis of Leptospira in urine should be considered.

Regarding *Rickettsia* spp. a study conducted by Kocher C. et al. between 2013 and 2014 in patients with undifferentiated fever in the Peruvian Amazon, found a frequency of 1.9% (38 of 2562 patients evaluated) of active rickettsia infections [39]. This contrasts with the results obtained by PCR in this paper, which found 8.2% of positives for *Rickettsia* spp. The age range of positive cases is similar in both studies. For many years, rickettsial disease was associated with higher rates in men and older people, however, recent studies did not show significant differences between gender or age, like our reported results [40, 41].

Although previous studies reported the presence of *Bartonella* spp. in the study region, and additionally several species of vectors have been reported and described [42, 43], in our study no case was identified. This could be due to various factors such as climate, competent distribution of the vector, and the presence or absence of a reservoir [44]. In this regard, climate can affect the transmission dynamics, geographic spread and re-emergence of vector-borne diseases through multiple pathways, including direct effects on the pathogen, the vector, non-human hosts and humans. Furthermore, climate change can alter entire ecosystem habitats, in which vectors or non-human hosts can thrive or fail [45].

In the matter of clinical characteristics of the disease the study concluded that there were no significant differences between the clinical presentation of patients distributed in groups of infected by each studied pathogen bacteria. But, regarding human leptospirosis, diverse clinical manifestations that could range from a mild acute fever and self-limited, to a severe and potentially fatal multi-organ failure, have been reported [44, 46]. Also, a variety of atypical manifestations and complications, which were not found during our study, have been described [47].

Approximately, 10% of patients infected by L*eptospira* spp. develop a severe disease [48]. The current paper showed a higher frequency of headache and myalgia in every group. The rest of the signs and symptoms were similar in all the age groups for each identified pathogen. In Brazil, regarding the clinical severity, an association between hospitalized patients with leptospirosis and in-creased mortality has been described [49]. The clinical severity should be understood as the presence of bleeding manifestations, alarm signs and signs of shock that are mentioned in Table 2.

Acute febrile illness caused by *Rickettsia* spp., is described in literature as transitory, non-severe, and with a heterogenous clinical presentation [50, 51]. In endemic regions, rapid diagnostic kits with high specificity and sensitivity are recommended for rickettsial infections. The rapid time response allows a timely diagnosis without the need to wait for serological results (seroconversion) or a long blood culture that could take between 10 days and 4 weeks [52, 53].

## Conclusions

*Rickettsia* and *Leptospira* have similar clinical presentations and are etiologies that should be considered within the differential diagnosis of undifferentiated AFI in the region studied. This is useful for physicians when choosing the appropriate empirical treatment. *B. bacilliformis* does not appear to play a major role in the etiologies of undifferentiated fever in this region. Vectors and reservoirs of *Rickettsia* and *Leptospira* should be a focus of attention in public health and disease control programs.

### Limitations

One of the main limitations of the study is the selection bias that limits the generalization of the results obtained and the extrapolation to other contexts. However, we consider that the number of samples analyzed is adequate to generate hypotheses for future rigorous and controlled investigations. Another major limitation is the lack of a control group.

## Data Availability

The data supporting the reported results are available from the corresponding author upon reasonable request.
